# Knowledge and compliance towards alendronate therapy among postmenopausal women with osteoporosis in Palestine

**DOI:** 10.1186/s12905-022-01690-5

**Published:** 2022-04-07

**Authors:** Asma Radwan, Naser Shraim, Josephean Elaraj, Anwar Hamad, Dana Fatayer, Bayan Jarar, Ayoub Johar, Areen Zriqah

**Affiliations:** Department of Pharmacy, College of Medicine and Health Sciences, An-Najah National University, P.O. Box 7, Nablus, Palestine

**Keywords:** Osteoporosis, Alendronate, Dosing instructions, Compliance, Palestine

## Abstract

**Background:**

Postmenopausal women compliance to alendronate therapy is suboptimal due to the complex dosing requirements. The poor compliance may increase their potential of fractures and the prevalence of side effects. In this study, the compliance of osteoporotic women on bisphosphonate therapy to the complex dosing instructions and their knowledge of alendronate-interactions were assessed.

**Methods:**

This is a cross-sectional study, using self-administered questionnaire involving 224 osteoporotic women on alendronate therapy, who visited the orthopedic clinics and community pharmacies in the West Bank. Data was collected using a validated questionnaire consisting of 4 sections and analyzed by descriptive statistics. Moreover, associations between patient's socio-demographic characteristics and the extent of compliance and knowledge of alendronate interactions are established in this study.

**Results:**

A total of 300 questionnaires were distributed and 224 were completed. The median compliance score to alendronate dosing instructions was 5 out of a possible maximum 7, and the median knowledge score about alendronate interactions was 7 out of a possible maximum 14. Factors found to affect either or both the knowledge and compliance to alendronate dosing instructions were, residency, and the source of instructions.

**Conclusion:**

This study identified the importance of compliance and knowledge gaps among postmenopausal women treated with alendronate. Therefore, appropriate knowledge about the importance of proper compliance to dosing instructions and avoidance of interactions is of a great benefit for maximizing clinical effectiveness, lowering fracture risk and prevention of adverse effects of alendronate among patients treated with alendronate in Palestine.

## Introduction

Osteoporosis is a bone degenerative disease affecting postmenopausal women, which is characterized by a reduction in bone mineral density with a consequent increase in bone fragility and risk of fractures [1].

Alendronate, which belongs to the bisphosphonates class, is one of the leading drugs for the treatment of osteoporosis [2]. This drug was proven to reduce the incidence of vertebral fractures by 40 to 50% [3]. However, it is characterized by low oral bioavailability (< 1%), complex administration instructions and specific interactions with food and other medications [4, 5]. Patient compliance to bisphosphonates therapy is suboptimal with discontinuation rates exceeding 30% in the first year [6–8]. Poor compliance of women to alendronate therapy is considered a problem that increases their potential of fractures and the prevalence of side effects [9, 10]. The experienced gastrointestinal (GI) adverse effects are the most common reason for the non-adherence as reported by 51.9% of women who stopped taking these medications [8, 11, 12]. In a large cohort study involving 9851 Italian postmenopausal women with osteoporosis, 19.1% of the patients taking daily or weekly alendronate discontinued the prescribed treatment within one year of therapy due to drugs related side effects [13]. Furthermore, complex dosing requirements are associated with patient’s poor compliance [14]. Alendronate dosing instructions (DIs) include: taking the medication in the morning with a full glass of plain water in upright position at least 30 min before breakfast or other medication. The failure to follow these instructions may result in treatment-related adverse events that further reduce compliance. Other factors associated with the poor compliance include the high cost of medication, poor understanding of the disease, patients’ un-willingness to be treated in an asymptomatic disease, and lack of motivation [15].

Food intake is likely to reduce alendronate bioavailability, which may lead to treatment failure. Black coffee, orange juice, mineral water and dairy products were shown to reduce the oral absorption of alendronate (by 60%). Furthermore, co administration of alendronate with other medications such as calcium supplements and antacids can lead to a loss in the efficacy of both medications [5]. Concomitant intake of non-steroidal anti-inflammatory drugs (NSAID) and corticosteroids may exacerbate the GI adverse effects. Unawareness of such interactions may reduce the therapeutic effectiveness of the drug and may contribute to the poor compliance. Therefore it is essential to assess patients’ knowledge about these interactions and their compliance to the dosing regimen.

Patient’s adherence is largely dependent on the information received from health care providers. Previous reports have addressed the importance of patient counseling in the compliance to bisphosphonates therapy. Pharmacist has a significant role in educating patients about the medication possible side effects and the proper dosing instructions [16–18]. The knowledge of pharmacists towards this information will lead to a success of the therapeutic process and improved patient compliance. In Palestine, pharmacists' knowledge toward the correct administration instructions of Alendronate, which is the only bisphosphonate available in the Palestinian market, was shown to be of a moderate level [19].

The main objectives of this work were to evaluate women’s compliance to the proper alendronate administration instructions, assess their knowledge about alendronate-food and other medications interactions, and measure the prevalence of alendronate adverse effects among patients with osteoporosis in Palestine.

## Methodology

### Study and sample design

This is a cross-sectional study using a self-administered questionnaire that was conducted in the time period between September 2017 and March 2018. The study involved 224 consecutive osteoporotic women receiving alendronate who attended ten orthopedic clinics and community pharmacies in the Northern area of the West Bank, which consist of 4 governorates: Nablus, Qalqiliah, Jenin and Tulkarem.

### Inclusion criteria

The eligibility criteria were as follows: (1) postmenopausal women being at least 45 years of age (the onset of menopause in females, after which an acceleration in the rate of bone loss was reported); (2) diagnosis of osteoporosis; (3) had been on the treatment with alendronate for at least 6 months (4) ability to understand the questions in order to help complete forms and questionnaires; and finally (5) willingness to participate in this study.

### The tools of the study

The questionnaire used was adapted and validated based on research objectives and survey instruments used in a previous published paper [18]. Furthermore, the questionnaire was evaluated and validated by a panel of experts from the Pharmacy Department at An-Najah University.

The questionnaire consists of four sections: The first section was about the socio-demographic and health characteristics: This section included personal descriptive data such as: age, years of education, residency, income as well as health characteristics such as, smoking concurrent medications and diseases. The questionnaire was available in Arabic language. The second section was about compliance with proper administration instructions: This section consisted of questions regarding the correct administration of alendronate on daily or weekly basis. These questions were dichotomous (yes/no) and multiple-choice questions. This section consisted of seven questions about participants’ practices regarding alendronate administration (timing of meal, timing of other drugs, amount of water intake, remaining in the upright position for 30 min, fasting before alendronate administration, whether it can be taken at the same time weekly or daily and what to do if the dose missed). One point is given to each question answered correctly and the summation of the points is labeled as compliance score to each participant. The third section was about the knowledge of alendronate interactions with food and other medications: In this section, patient's knowledge about alendronate food-drug interactions we assessed based on 14-items scale named knowledge score for each participant. Questions were asked about the possibility of co-administration of alendronate with certain medications, beverages and food (tap water, mineral water, milk, coffee, tea, dairy products, leafy vegetables, fruit juices, calcium supplements, vitamin D, antacids, antibiotics, cortisone and NSAIDs) or not. The fourth section was about the prevalence of adverse effects. This section includes the most common adverse effects that patient experienced during alendronate therapy.

### Data management and statistical analysis

Statistical analysis was performed using Statistical Package for Social Sciences (SPSS version 21). Data was expressed as means, medians and [25–75] percentiles for continuous variables and as frequencies (percentage) for categorical variables. For the comparative analysis, either Kruskal–Wallis or Mann-Whitney-U tests, as appropriate, were used to test significance between socio-demographic and clinical characteristics of participants. Normality of the data was tested by Kolmogorov–Smirnov test. The significance level was set at *P* ≤ 0.05.

### Ethics approval

The study was approved by the IRB committee of the College of Medicine and Health Sciences at An-Najah National University, Nablus, Palestine. The participants were asked to participate after explaining the nature and purpose of this study. A verbal informed consent was obtained from all the participants before administering the questionnaire.

## Results

### Socio-demographic and health characteristics related to the participants

A total of 224 of 300 surveys distributed were returned back (response rate 83%). The largest portion of the respondents was female with the age category (65–74 year) being the highest in frequency 27.7% (n = 62). The summary of other socio-demographic and health characteristics of the participants in this study is shown in Table 1.

**Table 1:** The socio-demographic and health characteristics of the participants

Variable	Alendronate use		
	Weekly (n = 214) % (N)	Daily (n = 10) % (N)	Total (n = 224) % (N)
*Age*	62.5		
45–54	23.4 (50)	70 (7)	25.4(57)
55–64	28.0 (60)	10 (1)	27.3(61)
65–74	28.0 (60)	20 (2)	27.7(62)
More than 75	20.6 (44)	0 (0)	19.6(44)
*Education level*			
Literate	24.3 (52)	0 (0)	23.2 (52)
Primary	25.7 (55)	20 (2)	25.4 (57)
Secondary	19.2 (41)	20 (2)	19.2 (43)
Diploma	13.6 (29)	30 (3)	14.3 (32)
Bachelor	15.9 (34)	30 (3)	16.5 (37)
Post graduate	1.4 (3)	0 (0)	1.3 (3)
*Income*			
Low	27.1 (58)	30 (3)	27.2 (61)
Moderate	68.7 (147)	50 (5)	67.9 (152)
High	4.2 (9)	20 (2)	4.9 (11)
*Residency*			
Urban	46.7 (100)	50 (5)	46.9 (105)
Rural	50.5 (108)	50 (5)	50.4 (113)
Camp	2.8 (6)	0 (0)	2.7 (6)
*Employment status*			
Employed	17.8 (38)	50 (5)	19.2 (43)
Unemployed	82.2 (176)	50 (5)	80.8 (181)
*Smoking*			
Current smoker	15.4 (33)	60 (6)	17.4 (39)
Previous smoker	4.7(10)	0 (0)	4.5(10)
Non-smoker	79.9 (171)	40 (4)	78.1 (175)
*Caffeine use*			
yes	70.1 (150)	80 (8)	70.5 (158)
No	29.9 (64)	20 (2)	29.5 (66)

The participants used alendronate for osteoporosis treatment. Most of the patients 95.5% (n = 214) reported using the weekly dose (70 mg). Only 4.5% (n = 10) of patients used the daily dose of alendronate. Almost all of the patients used concomitant medications along with alendronate, of which calcium supplements 86.2% (n = 193) and vitamin D 80.8% (n = 181) were the highest in frequencies, while 57.1% (n = 128) and 70.5% (n = 158) reported the use of antacids and NSAIDs concurrently with alendronate therapy, respectively.

### Compliance with the proper alendronate administration instructions

Most of the participants (83.9%) recalled receiving instructions on alendronate dosing regimen, its specific interactions and possible side effects. The sources of these instructions were from doctors (65.2%), pharmacists (40.2%), media (1.3%) and leaflets (9.4%), while 5.8% couldn't exactly remember their source of instructions. Furthermore, 63.4% of the participants stated reading the instructions, of whom 38.8% understood all of the instructions, while, 31.2% understood nothing.

The overall compliance of the participants was assessed based on their responses to the seven compliance questions. Table 2 shows the compliance for each dosing instruction among patients with regard to the dose used. The compliance of the patients received the drug on weekly basis was investigated separately than those received the daily dose. The median compliance score value for the sample was 5[4–6] out of a maximum possible score of 7. Overall, 42.6% of the participants had compliance score values 6–7. About 37.5%, 23.3% and 9.4% of participants were non-compliant with one, two or three of the dosing instructions, respectively.

**Table 2:** Compliance with the dosing instructions of alendronate

Dosing instructions of alendronate	Frequency of correct answer		
	Weekly (n = 214) N (%)	Daily (n = 10) N (%)	Total (n = 224) N (%)
Intake time is the same weekly or daily	184 (86)	10 (100)	194 (86.6)
Intake time is in the morning	187 (87.4)	5 (50)	192 (85.7)
Drinking large cup of water	153 (71.5)	4 (40)	157 (70.1)
Remain in the upright position for 30 min	179 (83.6)	4 (40)	183 (81.7)
What to do if missed dose	90 (42.1)	4 (50)	76 (33.9)
Timing of meal with regard to alendronate	155 (72.4)	1 (10)	164 (74.3)
Timing between alendronate and other medications	123 (57.5)	1 (10)	124 (55.4)

The association between the different socio-demographic and health characteristics of the patients and their compliance score is shown in Table 3. A significant difference in the compliance score values was found among participants according to the residency and the source of instructions received. Patients who recalled receiving instructions had relatively higher compliance score values than those who didn't (Table 4)**.** Furthermore, respondents who had received instructions from pharmacists had higher scores values than those from other sources (*P* = 0.025). Moreover, participants who reported reading the instructions and understanding all of these instructions had higher compliance score values.

**Table 3:** The association between the socio-demographic characteristics and the compliance and knowledge scores

Variable	Compliance score				Knowledge score	
	Weekly (n = 214)		Total (n = 224)		Total (n = 224)	
	Inter quartiles Median	*P*-value	Inter quartiles Median	*P*-value	Inter quartiles Median	*P*-value
*Age*		0.124		0.104		0.374
45–54	5 (5–6)		5 (5–6)		6.5 (4–9)	
55–64	5 (4–5)		5 (4–5)		7 (3–10)	
65–74	5 (4–6)		5 (4–6)		7 (5–9)	
More than 75	5 (4–6)		5 (4–6)		10 (6–11.75)	
*Education level*		0.11		0.253		0.320
Literate	5 (3–6)		5 (3–6)		8 (5–9.75)	
Primary	6 (4–6)		6 (4–6)		7 (3–10)	
Secondary	5 (3–6)		5 (3–6)		7 (3–9)	
Diploma	5 (4–6)		5 (4–6)		6.5 (3.25–8.75)	
Bachelor	5 (4–6)		5.5 (4–6)		5 (7–9)	
Post graduate	6 (4–6)		6 (4–6)		4 (9–11)	
*Income*		0.582		0.528		0.01
Low	5 (4–6)		5 (3.7–6)		8 (5–10)	
Moderate	5 (4–6)		5 (4–6)		7 (4–9)	
High	4 (4–6)		6 (4–6)		7 (4–12)	
*Residency*		0.014		0.029		0.290
Urban	6 (4–6)		6 (4–6)		7 (4–9)	
Rural	5 (4–6)		5 (4–6)		8 (4–10)	
Camp	2.5 (1–5.25)		2.5 (1–5.25)		6.5 (3.25–11.25	
*Employment status*		0.055		0.034		0.704
Employed	5 (4–6)		5 (4–6)		8 (5–10)	
Unemployed	5 (4–6)		5 (4–6)		7 (3.5–10)	
*Smoking*		0.204		0.087		0.650
Current smoker	5 (3–6)		5 (3–6)		6 (4–8)	
Previous smoker	6 (5.7–6)		6 (5.7–6)		9 (4.25–10)	
Non-smoker	5 (4–6)		5 (4–6)		7 (4–10)	
*Caffeine use*		0.155		0.180		0.537
yes	5 (4–6)		5 (4–6)		7 (4–10)	
No	6 (4–6)		6 (4–6)		7 (3–9)

**Table 4:** The association between the source of dosing instructions and the compliance and knowledge scores

Variable	Compliance score		Knowledge score	
	Inter quartiles Median	*P*-value	Inter quartiles Median	*P*-value
Receiving instructions	5 (5–6)	0.001	8 (4–11)	0.033
Not receiving instructions	4 (3–5)	0.744	7 (4.25–9)	0.122
*Source of instructions*				
Doctor	5 (5–6)	0.498	8 (4–11)	0.116
Pharmacist	5 (5–6)	0.025	9 (5–11)	0.007
Media	5.5 (5–6)	0.499	7.5 (5–10.75)	0.774
Leaflets	5 (4–6)	0.964	6 (3–9)	0.291
Unknown	4 (2.5–5)	0.001	5 (2–8.5	0.074
Read the instructions	5 (5–6)	0.002	8 (5–11)	0.001
*Understand the instructions*		0.001		0.001
All	6 (5–6)		9 (6–11)	
Some	5 (5–6)		6 (5–9)	
Little	4 (2–5.5)		6 (4–8)	

### Knowledge about alendronate food-drug interactions

The overall knowledge of the study participants was assessed based on their responses to the fourteen questions about alendronate food-drug interactions as can be seen in Table 5. The values reported for each question represent the percentage of the correct answers for each type of food or medication. The median knowledge score value was 7 [4–10] from a maximum possible value of 14. In the section of food—alendronate interactions, 86.6% (n = 194) of the respondents believed that alendronate could be taken with tap water; however, most of them 67.9% (n = 152) did not know that it shouldn’t be administered with mineral water. Participants scored high to the question about the interaction of alendronate with tea and coffee compared to other types of food. With respect to alendronate- drug interactions, the majority of participants were not aware that alendronate couldn’t be concomitantly administered with calcium supplements, NSAIDs, antacids or corticosteroids.

**Table 5:** Knowledge of the possible food and medications interactions of alendronate

Food or beverages	Correct answers (n = 224) N (%)
*Is it possible to co administer alendronate with the following*	
Tap water	194 (86.6)
Mineral water	72 (32.1)
Milk	137 (61.2)
Coffee	163 (72.8)
Tea	160 (71.4)
Dairy products	137 (61.2)
Leafy vegetables	118 (52.7)
Fruit juices	103 (46.0)
*Medications*	
Calcium supplements	62 (27.7)
Vitamin D	115 (51.3)
Antacids	94 (43.3)
Cortisone	88 (40.6)
NSAIDS	69 (30.8)

The association between the different socio-demographic and health characteristics of the patients and their knowledge score is shown in Table 3. A significant effect of the income level and the source of instruction received on the knowledge score value was found among the participants.

### Side effects experienced by the patients

The most common side effect reported was GI intolerance. 43.8% (n = 98) of the participants experienced gastritis, 24.1% (n = 54) had peptic ulcer, where 25% experienced dyspepsia and hemoptysis 4.9%. Others reported side effects were dysphagia, painful and swollen gums.

## Discussion

Compliance with alendronate therapy has shown to be difficult. In this study, only 42% of the participants were compliant with almost all of the dosing instructions, whereas, 51.6% had compliance score values 3–5. About 6.4% of the participants were compliant with only one or two of the dosing instructions. The extent of compliance to dosing instructions among participants was comparable to previous reports. A previous study that assessed patient's compliance with dosing instructions of weekly dosed bisphosphonates, pointed that only 44% of respondents were compliant with all DIs. Compliance with DIs related to staying upright was 71% compared to 81.7% in this study [18]. In a study by Ettinger et al. [8], about 55.8% of the women didn't comply with at least one dosing instruction regarding alendronate administration. Moreover, more than half of them failed to comply with instructions regarding food, drinks and medications [8]. Similarly, in this study the compliance of participants with DIs regarding time of meal and medications was 55.4%. About 18% of the participants failed to comply with remaining in the upright position 30 min after taking the drug compared to 13.5% in previous reports.

These findings suggest that residency is one the factors associated with patient's compliance. A previous study found that compliance with DIs associated positively with education [20]. However, in this study, education level has no significant effect on patient’s compliance.

Regarding the patients' knowledge of alendronate interactions**,** the mean knowledge score value for this sample was 7.0. Providing patients with information about the proper dosing instructions and adverse effects is associated with higher knowledge and compliance score values [20]. In this study, 16% the participants did not recall being received instructions on alendronate dosing regimen and specific interactions. These finding is similar to Hamilton et al. results, about 15% of patients taking risedronate reported not receiving an instruction leaflet [21]. Patients who recalled receiving and understanding of instructions were more likely to have good compliance and knowledge. These findings are consistent with a previous study [8]. Furthermore, the source of instructions received has an effect. Respondents received instructions from pharmacists had higher compliance to dosing instructions and had higher knowledge of interactions. These finding suggest the role of pharmacists in counseling patients [17]. Similar results were found in a study conducted in Malaysia, which indicated that pharmacists have a role in improving medication adherence to alendronate [16].

Furthermore, reading and understanding the instructions leaflets contribute to higher compliance with dosing instructions. These results suggest the necessity to improve consumers' understanding of leaflet instructions. Principle methods to achieve this goal include instructing consumers to intentionally read the entire drug leaflet content every time when purchasing or taking drugs. Also, using pictograms as much as possible in drug' leaflet is also a suggestion.

The largest portion of our sample patients recalled receiving four or more medications along with alendronate. High portion of patients used calcium supplements, antacids NSIADs and glucocorticoids concurrently with alendronate. NSAIDs can exacerbate gastrointestinal adverse effects. In addition, considerable portion of respondents used NSAIDs to treat musculoskeletal pain that they experienced during alendronate therapy. As a result, 57.2% of the participants reported that they have consumed antacids concurrently with alendronate therapy to relieve GI symptoms caused by co administration of alendronate with NSAIDs.

The high incidence of the experienced side effects is linked to the inappropriate use of alendronate or the concurrent use of other drugs. Patients who had higher compliance and knowledge score values were found to experience side effects to a lesser extent than those who had lower score values. GI adverse effects were the most frequent among patients.

Accordingly, strategies should be implemented to ensure optimal women compliance which include: providing adequate information for patients about alendronate specific dosing instructions, interactions and side effects as well as developing educational programs for patients and health care providers to improve patients’ counseling to the proper administration of alendronate.

### The limitations of this study

The limitations of this study include: determination of the appropriate sample size due to the lack of prior research studies on alendronate use prevalence in Palestine. Moreover, the answers reported by the respondent cannot be validated and recall bias is possible, which cannot be avoided in survey studies.

## Conclusions

In conclusion, postmenopausal women compliance to specific dosing instructions of alendronate medication and knowledge of alendronate specific food and drugs interactions is suboptimal and requires more attention. The high prevalence of alendronate side effects can contribute to the suboptimal adherence to proper instruction. GI side effects are is the most common adverse effects. Factors found to have significant effect on knowledge and compliance score values were; age, education level, residency, number of chronic medications.

There is a need to improve the compliance and increase the awareness of alendronate interactions and the follow up of the patients by health care providers and the implementation of strategies to overcome barriers for adherence.

## Data Availability

The raw data supporting the findings presented in the current study will be available from the corresponding author upon request.
